# Supplementary material for: Risk factors for increased COVID-19 case-fatality in the United States: A county-level analysis during the first wave
Source: PLoS One. 2021 Oct 14;16(10):e0258308. doi: 10.1371/journal.pone.0258308 (PMC8516194; doi:10.1371/journal.pone.0258308)
Supplement: S1 Appendix — (PDF) [file pone.0258308.s001.pdf]

Appendix

PAGE

Appendix 1: Study population flow chart 2

Appendix 2: Justifications for variable inclusion 3

Appendix 3: Variables and data sources 13

Appendix 4: Descriptive statistics for county variables retained for analysis 20

Appendix 5: Regression results 21

Appendix 6: Two variable quantile stratifications 22

Appendix References 29

Appendix 1: Study population flow chart

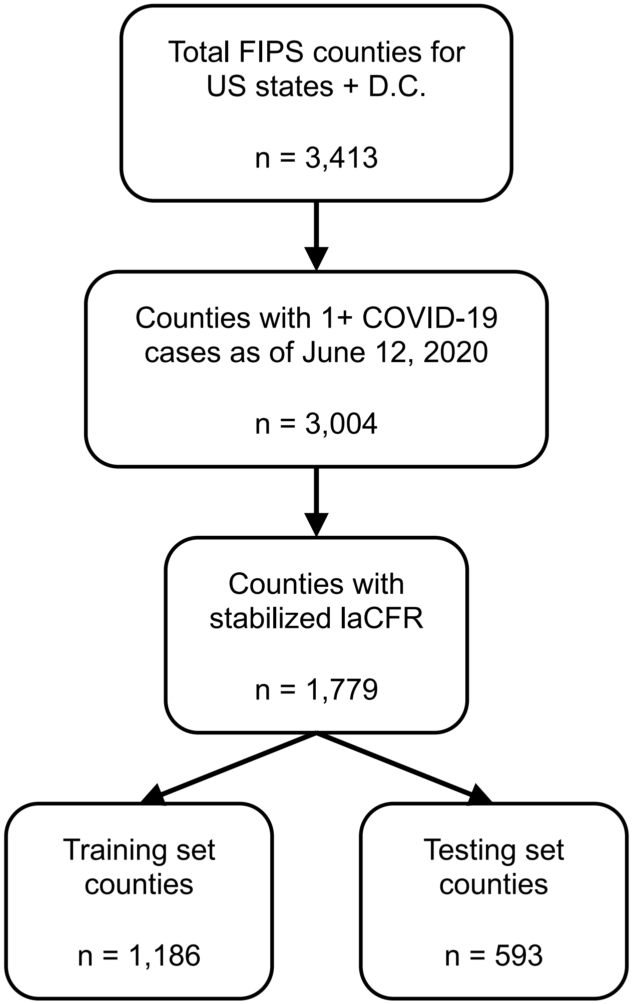

Figure A1. Study population flow chart of counties included in the analysis.

Appendix 2: Justifications for variable inclusion

Variable Topic	Variable Code	Variable Description	Justifications
Comorbidities	como_allheartdis_hosp	Heart Disease Hospitalization Rate per 1,000 Medicare Beneficiaries, 65+	This variable is associated with the severity of and mortality due to COVID-19.[1]
Comorbidities	como_allheartdis_mort	Heart Disease Death Rate per 100,000, 35+	This variable is associated with the severity of and mortality due to COVID-19.[1]
Comorbidities	como_asthma	Age-adjusted prevalence of adults who have been told they currently have asthma	This variable is associated with the severity of and mortality due to COVID-19.[2]
Comorbidities	como_cancer5yr	Age adjusted incidence of all cancer-5-year prevalence	This variable is associated with the severity of and mortality due to COVID-19.[3]
Comorbidities	como_COPD	Age-adjusted prevalence of adults diagnosed with chronic obstructive pulmonary disease	This variable is associated with the severity of and mortality due to COVID-19.[4]
Comorbidities	como_cvd_hosp	Total Cardiovascular Disease Hospitalization Rate per 1,000 Medicare Beneficiaries, 65+	This variable is associated with the severity of and mortality due to COVID-19.[1]
Comorbidities	como_cvd_mort	Total Cardiovascular Disease Death Rate per 100,000, All Ages	This variable is associated with the severity of and mortality due to COVID-19.[1]
Comorbidities	como_pdiabetes	Age-adjusted prevalence of adults aged 20+ years with diagnosed diabetes (in %) by county	This variable is associated with the severity of and mortality due to COVID-19.[5]
Comorbidities	como_htn_hosp	Hypertension Hospitalization Rate per	This variable is associated with the severity of and mortality due to COVID-19.[6]

		1,000 Medicare Beneficiaries, 65+	
Comorbidities	como_htn_mort	Hypertension Death Rate per 100,000 (any mention), 35+	This variable is associated with the severity of and mortality due to COVID-19.[6]
Comorbidities	como_ medicareheartdizprev	Prevalence (in %) of heart disease among Medicare beneficiaries	This variable is associated with the severity of and mortality due to COVID-19.[1]
Comorbidities	como_pobesity	Age-adjusted prevalence of adults aged 20+ years with obesity (in %) by county	This variable is associated with the severity of and mortality due to COVID-19.[7]
Comorbidities	como_smoking	Age-adjusted prevalence of adults who are current smokers (variable calculated from one or more BRFSS questions)	This variable is associated with the severity of and mortality due to COVID-19.[4]
Comorbidities	como_stroke_hosp	Stroke Hospitalization Rate per 1,000 Medicare Beneficiaries, 65+	This variable is associated with the severity of COVID-19.[8]
Comorbidities	como_stroke_mort	Stroke Death Rate per 1,000, 35+	This variable is associated with the severity of COVID-19.[8]
Demographics	demo_landarea	County land area in square meters	Historically, more rural areas saw a lower burden of infectious disease because smaller populations meant diseases were less likely to be circulating,[9] suggesting that counties with smaller populations or larger land areas may be less impacted if those who are unwell come into contact with fewer people allowing the disease to burn out before cases and CFR climb.
Demographics	demo_p60more	Percentage of population aged 60 years or older	COVID-19 has a higher fatality rate for older populations, while sparing younger ages from more severe forms of the disease.[10]

Demographics	demo_p65more	Percentage of population aged 65 years or older	COVID-19 has a higher fatality rate for older populations, while sparing younger ages from more severe forms of the disease.[10]
Demographics	demo_p45_64	Percentage of population aged 45 to 64	COVID-19 has a higher fatality rate for older populations, while sparing younger ages from more severe forms of the disease.[10]
Demographics	demo_popdensity	Population density	Population density may make social distancing more challenging and may also result in a higher effective contact rate.[11]
Demographics	demo_population	Total population of each county (same as demo_bridgedrace_total)	Historically, more rural areas saw a lower burden of infectious disease because smaller populations meant diseases were less likely to be circulating,[9] suggesting that counties with smaller populations or larger land areas may be less impacted if those who are unwell come into contact with fewer people allowing the disease to burn out before cases and CFR climb
Healthcare access & capacity	hc_hospitals	Number of Hospitals	Counties with greater healthcare resources available will presumably be able to manage a higher case-load before becoming overwhelmed.[12]
Healthcare access & capacity	hc_hospitals_per10000	Number of Hospitals per 10000	Counties with greater healthcare resources available will presumably be able to manage a higher case-load before becoming overwhelmed.[12]
Healthcare access & capacity	hc_icubeds_per1000	Number of ICU beds per 1000	Counties with greater healthcare resources available will presumably be able to manage a higher case-load before becoming overwhelmed.[12]
Healthcare access & capacity	hc_icubeds	Number of ICU beds per 1000	Counties with greater healthcare resources available will presumably be able to manage a higher case-load before becoming overwhelmed.[12]
Healthcare access & capacity	hc_icubeds_per60more	Number of ICU beds per >60 year resident	Counties with greater healthcare resources available will presumably be able to manage a higher case-load before becoming overwhelmed.[12]

Healthcare access & capacity	hc_icubeds_per65more	Number of ICU beds per >65 year resident	Counties with greater healthcare resources available will presumably be able to manage a higher case-load before becoming overwhelmed.[12]
Healthcare access & capacity	hc_medicaid	Medicaid eligible	Uninsured Americans are less likely to access health care when needed, more likely to delay treatment, are at higher risk of hospitalization, and also more likely to have preventable illnesses or uncontrolled chronic illnesses, which may put them at higher risk of serious COVID-19 illness.[13,14]
Healthcare access & capacity	hc_Pnotinsured_acs	Percentage without Health Insurance	Uninsured Americans are less likely to access health care when needed, more likely to delay treatment, are at higher risk of hospitalization, and also more likely to have preventable illnesses or uncontrolled chronic illnesses, which may put them at higher risk of serious COVID-19 illness.[13,14]
Healthcare access & capacity	hc_primarycare	Number of primary care physicians in the county	Counties with greater healthcare resources available will presumably be able to manage a higher case-load before becoming overwhelmed.[12]
Healthcare access & capacity	hc_primarycare_per1000	Primary Care Physician per capita	Counties with greater healthcare resources available will presumably be able to manage a higher case-load before becoming overwhelmed.[12]
Non-pharmaceutical intervention	npi_keystone_closing_of_public_venues	Government order closing venues such as restaurants, theaters, and bars	We expect non-pharmaceutical intervention (NPI) can have an influence on COVID-19 mortality. At the time of variable inclusion, there were no published results for COVID-19 NPI yet.
Non-pharmaceutical intervention	npi_keystone_gathering_size_10_0	Gathering size limited to 10 or fewer people	We expect non-pharmaceutical intervention (NPI) can have an influence on COVID-19 mortality. At the time of variable inclusion, there were no published results for COVID-19 NPI yet.
Non-pharmaceutical intervention	npi_keystone_gathering_size_100_to_26	Gathering size limited to 26 to 100 people	We expect non-pharmaceutical intervention (NPI) can have an influence on COVID-19 mortality. At the time of variable inclusion, there were no published results for COVID-19 NPI yet.

Non-pharmaceutical intervention	npi_keystone_gathering_size_25_to_11	Gathering size limited to 11 to 25 people	We expect non-pharmaceutical intervention (NPI) can have an influence on COVID-19 mortality. At the time of variable inclusion, there were no published results for COVID-19 NPI yet.
Non-pharmaceutical intervention	npi_keystone_gathering_size_500_to_101	Gather size limited to 101 to 500 people	We expect non-pharmaceutical intervention (NPI) can have an influence on COVID-19 mortality. At the time of variable inclusion, there were no published results for COVID-19 NPI yet.
Non-pharmaceutical intervention	npi_keystone_lockdown	Lockdown	We expect non-pharmaceutical intervention (NPI) can have an influence on COVID-19 mortality. At the time of variable inclusion, there were no published results for COVID-19 NPI yet.
Non-pharmaceutical intervention	npi_keystone_non_essential_services_closure	Government order closing non-essential services and shops	We expect non-pharmaceutical intervention (NPI) can have an influence on COVID-19 mortality. At the time of variable inclusion, there were no published results for COVID-19 NPI yet.
Non-pharmaceutical intervention	npi_keystone_Other	Other, unspecified, NPI	We expect non-pharmaceutical intervention (NPI) can have an influence on COVID-19 mortality. At the time of variable inclusion, there were no published results for COVID-19 NPI yet.
Non-pharmaceutical intervention	npi_keystone_religious_gatherings_banned	Cancellation of religious gatherings either explicitly or implicitly through gathering size limitations that do not exempt religious services	We expect non-pharmaceutical intervention (NPI) can have an influence on COVID-19 mortality. At the time of variable inclusion, there were no published results for COVID-19 NPI yet.
Non-pharmaceutical intervention	npi_keystone_school_closure	Closure of schools and university	We expect non-pharmaceutical intervention (NPI) can have an influence on COVID-19 mortality. At the time of variable inclusion, there were no published results for COVID-19 NPI yet.
Non-pharmaceutical intervention	npi_keystone_shelter_in_place	An order indicating that people should shelter in	We expect non-pharmaceutical intervention (NPI) can have an influence on COVID-19 mortality. At

		their homes except for essential reasons	the time of variable inclusion, there were no published results for COVID-19 NPI yet.
Non-pharmaceutical intervention	npi_keystone_social_distancing	Social distancing mandate of at least 6' between people	We expect non-pharmaceutical intervention (NPI) can have an influence on COVID-19 mortality. At the time of variable inclusion, there were no published results for COVID-19 NPI yet.
Socioeconomics	ses_hhincome	Median household income in the past 12 months	For most racial groups, increased income correlates with improved health.[15]
Socioeconomics	ses_pnohighschool	Percentage of no high school diploma by county	There is an established association of lower educational attainment and poorer health, including chronic illness and mortality,[16] along with the importance of high school education as a measure[17] of this association.
Socioeconomics	ses_ppoverty	Percentage of residents with income in the past 12 months below poverty level by county	For most racial groups, increased income correlates with improved health.[15]
Socioeconomics	ses_punemployed	Percentage of unemployed (not in labor force) by county	Unemployment is associated with poor health,[18] and also contributes to homelessness,[19] which is, in turn, a risk for COVID-19 infection.[20]
Social vulnerability	sv_groupquarterspop	Number of persons in institutionalized group quarters	This variable is used to construct the Social Vulnerability Index (SVI). Group living arrangements represent increased risk of SARS-CoV-2 transmission due to both difficulties in maintaining hygiene and social distancing in these settings and due to the risk that caregivers who visit multiple homes have an increased risk of both acquiring and spreading the virus.[21]
Social vulnerability	sv_p17below	Age 17 or younger	This variable is used to construct the Social Vulnerability Index (SVI). COVID-19 has a higher fatality rate for older populations, while sparing younger ages from more severe forms of the disease.[10]

Social vulnerability	sv_pcrowding	Percentage of occupied housing units with more people than rooms	This variable is used to construct the Social Vulnerability Index (SVI). This variable represents increased challenges to social distancing. For instance, an individual falling ill in a crowded apartment will have more difficulty in self-isolating than someone living in a spacious home. Individuals living in apartment complexes will have more difficulty in maintaining a 6-foot distance when outside than individuals with access to backyards.[22,23]
Social vulnerability	sv_pdisability	Older than age 5 with disability	This variable is used to construct the Social Vulnerability Index (SVI). This variable represents barriers to healthcare access and increased likelihood of greater health needs and worse outcomes from existing health conditions.[24]
Social vulnerability	sv_penglish	Percentage of population (age 5+) who speak English "less than well"	This variable is used to construct the Social Vulnerability Index (SVI). Populations with poor English skills are likely to have increased difficulty in accessing accurate health information and decreased visits to healthcare professionals.[25]
Social vulnerability	sv_pminority	Percentage minority (not non-Hispanic White)	This variable is used to construct the Social Vulnerability Index (SVI). The percentage of the county population who belongs to the racial minority is included for the same reason that different races/ethnicities were included as part of the demographic data above (Essentially, this is a grouping that includes all except non-Hispanic White. It can be understood as it might be better to use a composite variable to increase statistical power). Historically, more rural areas saw a lower burden of infectious disease because smaller populations meant diseases were less likely to be circulating,[9] suggesting that counties with smaller

			populations or larger land areas may be less impacted if those who are unwell come into contact with fewer people allowing the disease to burn out before cases and CFR climb.
Social vulnerability	sv_pmobilehome	Percentage of total housing units with mobile home	This variable is used to construct the Social Vulnerability Index (SVI). This variable represents increased challenges to social distancing. For instance, an individual falling ill in a crowded apartment will have more difficulty in self-isolating than someone living in a spacious home. Individuals living in apartment complexes will have more difficulty in maintaining a 6-foot distance when outside than individuals with access to backyards.[18,19]
Social vulnerability	sv_pmultiunit	Percentage of total housing units with 10 or more units	This variable is used to construct the Social Vulnerability Index (SVI). This variable represents increased challenges to social distancing. For instance, an individual falling ill in a crowded apartment will have more difficulty in self-isolating than someone living in a spacious home. Individuals living in apartment complexes will have more difficulty in maintaining a 6-foot distance when outside than individuals with access to backyards.[18,19]
Social vulnerability	sv_pnovehicle	Percentage of households with no vehicle available	This variable is used to construct the Social Vulnerability Index (SVI). Increased reliance on public transport will create more crowded transport and a higher risk of transmission.[26]
Social vulnerability	sv_singleparent	Single-parent household with children under 18	This variable is used to construct the Social Vulnerability Index (SVI). These households are likely to experience increased difficulties finding childcare. The potential impact on absenteeism for

			healthcare workers could lead to higher mortality rates[27] if more of the workforce are single parents.
Demographics	demo_bridgedrace_p_ american_indians_alaskan	Percentage of (non Hispanic) American Indian or Alaska Native	In the US structural racism leads to racial/ethnic populations' lack of access to health care and receipt of low-quality health care, contributing to substantial health disparities,[28] which may, in turn, result in worse outcomes for COVID-19 patients.
Demographics	demo_bridgedrace_p_ asians_pacific	Percentage of (non-Hispanic) Asian or Pacific Islander.	In the US structural racism leads to racial/ethnic populations' lack of access to health care and receipt of low-quality health care, contributing to substantial health disparities,[28] which may, in turn, result in worse outcomes for COVID-19 patients.
Demographics	demo_bridgedrace_p_ blacks	Percentage of (non-Hispanic) Black or African American	In the US structural racism leads to racial/ethnic populations' lack of access to health care and receipt of low-quality health care, contributing to substantial health disparities,[28] which may, in turn, result in worse outcomes for COVID-19 patients.
Demographics	demo_bridgedrace_p_hisp	Percentage of Hispanic or Latino	In the US structural racism leads to racial/ethnic populations' lack of access to health care and receipt of low-quality health care, contributing to substantial health disparities,[28] which may, in turn, result in worse outcomes for COVID-19 patients.
Demographics	demo_bridgedrace_p_ whites	Percentage of (non-Hispanic) White	In the US structural racism leads to racial/ethnic populations' lack of access to health care and receipt of low-quality health care, contributing to substantial health disparities,[28] which may, in turn, result in worse outcomes for COVID-19 patients.
Demographics	demo_bridgedrace_total	Total population of each county (same as demo_population)	Historically, more rural areas saw a lower burden of infectious disease because smaller populations meant diseases were less likely to be circulating,[9] suggesting that counties with smaller populations or larger land areas may be less impacted if those who are unwell come into contact with fewer

			people allowing the disease to burn out before cases and CFR climb
Time	days_since_first_case	Number of days between first detected COVID19 case and final date of included case data, New York City data by county from NYC public health website, Kansas City counties were excluded	We expect this variable can have an influence on COVID-19 mortality. At the time of variable inclusion, there were no published results for this variable yet.

Appendix 3: Variables and data sources

Model Inclusion Status	Variable Code	Level Data	Data Source	Year(s) Collected	Variable Unit Description
Included, linking variable	FIPS	County	US Census TIGER shapefile	2018	ID number
Excluded, highly correlated	como_allheartdis_hosp	County	CDC, Interactive Atlas of Heart Disease and Stroke	2016-2018	Incidence per 1000, 65+
Excluded, highly correlated	como_allheartdis_mort	County	CDC, Interactive Atlas of Heart Disease and Stroke	2014-2016	Incidence per 100,000
Included in final model	como_asthma	State	BRFSS	2018	Adjusted prevalence %
Excluded, non-significant in multivariate model	como_cancer5yr	County if available, o/w State	NIH, National Cancer Institute, State Cancer Profiles	2012-2016	5-year incidence
Excluded, highly correlated	como_COPD	State	BRFSS	2018	Adjusted prevalence %
Excluded, highly correlated	como_cvd_hosp	County	CDC, Interactive Atlas of Heart Disease and Stroke	2016-2018	Incidence per 1000, ages 65+
Excluded, highly correlated	como_cvd_mort	County	CDC, Interactive Atlas of Heart Disease and Stroke	2016-2018	Incidence per 100,000
Excluded, non-significant in bivariate model	como_pdiabetes	County	CDC, US Diabetes Surveillance System	2016	Percentage (%)
Excluded, highly correlated	como_htn_hosp	County	CDC, Interactive Atlas of Heart Disease and Stroke	2016-2018	Incidence per 1000, 65+
Excluded, non-significant in bivariate model	como_htn_mort	County	CDC, Interactive Atlas of Heart Disease and Stroke	2016-2018	Incidence per 1000
Excluded, non-significant in multivariate model	como_ medicareheartdizprev	County	CDC, Interactive Atlas of Heart Disease and Stroke	2018	Prevalence per 1000, medicare beneficiaries

Excluded, highly correlated	como_pobesity	County	CDC, Interactive Atlas of Heart Disease and Stroke	2016	Percentage (%)
Excluded, non-significant in bivariate model	como_smoking	State	BRFSS	2018	Age adjusted prevalence
Excluded, highly correlated	como_stroke_hosp	County	CDC, Interactive Atlas of Heart Disease and Stroke	2018	Incidence per 1000
Excluded, highly correlated	como_stroke_mort	County	CDC, Interactive Atlas of Heart Disease and Stroke	2014-2016	Incidence per 1000
Excluded, non-significant in bivariate model	demo_landarea	County	US Census TIGER shapefile	2018	Area in square kilometer
Excluded, highly correlated	demo_p60more	County	Bridged race	2010-2018 average	Percentage (%)
Included in final model	demo_p65more	County	Bridged race	2010-2018 average	Percentage (%)
Excluded, highly correlated	demo_p45_64	County	Bridged race	2010-2018 average	Percentage (%)
Excluded, highly correlated	demo_popdensity	County	Bridged race	2018	Person per square kilometer
Excluded, highly correlated	demo_population	County	Bridged race	2018	Count
Included in final model	hc_hospitals	County	CDC, Interactive Atlas of Heart Disease and Stroke and demo_population variable	2018	Total number of hospitals in county
Included in final model	hc_hospitals_per10000	County	CDC, Interactive Atlas of Heart Disease and Stroke and demo_population variable	2018	Number of hospitals per 10,000 people in county
Excluded, highly correlated	hc_icubeds_per1000	County	Kaiser Health News analysis of hospital cost reports filed to the Centers for Medicare	2018/2019	Number per 1000 persons in the county

			& Medicaid Services American Community Survey, (5-year estimate)		
Excluded, highly correlated	hc_icubeds	County	Kaiser Health News analysis of hospital cost reports filed to the Centers for Medicare & Medicaid Services American Community Survey, (5-year estimate)	2018/2019	Number of ICU beds in county
Excluded, non-significant in multivariate model	hc_icubeds_per60more	County	Kaiser Health News analysis of hospital cost reports filed to the Centers for Medicare & Medicaid Services	2018/2019	Number per 1000 persons aged 60+
Excluded, highly correlated	hc_icubeds_per65more	County	Kaiser Health News analysis of hospital cost reports filed to the Centers for Medicare & Medicaid Services	2018/2019	Number per 1000 persons aged 65+
Excluded, highly correlated	hc_medicaid	County	CDC, Interactive Atlas of Heart Disease and Stroke	2018	Percentage (%)
Included in final model	hc_Pnotinsured_acs	County	US Census American Community Survey 5-Year Data	2018	Percentage (%)
Excluded, non-significant in bivariate model	hc_primarycare	County	Health Resources and Services Administration, (Area Health Resources File)	2016	Count
Excluded, highly correlated	hc_primarycare_per1000	County	Health Resources and Services Administration, (Area Health Resources File)	2016	Adjusted incidence rate per 1000
Excluded, highly correlated	npi_keystone_closing_of_public_venues	County	KeyStone Coronavirus City and County Non-Pharmaceutical Intervention Rollout Date Dataset	2020	Date

Excluded, highly correlated	npi_keystone_gathering_size_10_0	County	KeyStone Coronavirus City and County Non-Pharmaceutical Intervention Rollout Date Dataset	2020	Date
Excluded, highly correlated	npi_keystone_gathering_size_100_to_26	County	KeyStone Coronavirus City and County Non-Pharmaceutical Intervention Rollout Date Dataset	2020	Date
Excluded, highly correlated	npi_keystone_gathering_size_25_to_11	County	KeyStone Coronavirus City and County Non-Pharmaceutical Intervention Rollout Date Dataset	2020	Date
Excluded, highly correlated	npi_keystone_gathering_size_500_to_101	County	KeyStone Coronavirus City and County Non-Pharmaceutical Intervention Rollout Date Dataset	2020	Date
Excluded, highly correlated	npi_keystone_lockdown	County	KeyStone Coronavirus City and County Non-Pharmaceutical Intervention Rollout Date Dataset	2020	Date
Excluded, highly correlated	npi_keystone_non_essential_services_closure	County	KeyStone Coronavirus City and County Non-Pharmaceutical Intervention Rollout Date Dataset	2020	Date
Excluded, highly correlated	npi_keystone_Other	County	KeyStone Coronavirus City and County Non-Pharmaceutical Intervention Rollout Date Dataset	2020	Date
Included in final model	npi_keystone_religious_gatherings_banned	County	KeyStone Coronavirus City and County Non-Pharmaceutical Intervention Rollout Date Dataset	2020	Date

Excluded, highly correlated	npi_keystone_school_closure	County	KeyStone Coronavirus City and County Non-Pharmaceutical Intervention Rollout Date Dataset	2020	Date
Excluded, highly correlated	npi_keystone_shelter_in_place	County	KeyStone KeyStone Coronavirus City and County Non-Pharmaceutical Intervention Rollout Date Dataset	2020	Date
Excluded, non-significant in multivariate model	npi_keystone_social_distancing	County	KeyStone Coronavirus City and County Non-Pharmaceutical Intervention Rollout Date Dataset	2020	Date
Excluded, highly correlated	ses_hhincome	County	US Census American Community Survey 5-Year Data	2018	Median income in US Dollars
Excluded, highly correlated	ses_pnohighschool	County	US Census American Community Survey 5-Year Data	2018	Percentage (%)
Excluded, highly correlated	ses_ppoverty	County	US Census American Community Survey 5-Year Data	2018	Percentage (%)
Excluded, non-significant in multivariate model	ses_punemployed	County	US Census American Community Survey 5-Year Data	2018	Percentage (%)
Excluded, highly correlated	sv_groupquarterspop	County	US Census American Community Survey 5-Year Data	2018	Count
Excluded, highly correlated	sv_p17below	County	US Census American Community Survey 5-Year Data	2018	Percentage (%)

Excluded, non-significant in multivariate model	sv_pcrowding	County	US Census American Community Survey 5-Year Data	2018	Percentage (%)
Excluded, highly correlated	sv_pdisability	County	US Census American Community Survey 5-Year Data	2018	Percentage (%)
Excluded, highly correlated	sv_penglish	County	US Census American Community Survey 5-Year Data	2018	Percentage (%)
Excluded, highly correlated	sv_pminority	County	CDC SVI	2018	Percentage (%)
Included in final model	sv_pmobilehome	County	US Census American Community Survey 5-Year Data	2018	Percentage (%)
Excluded, highly correlated	sv_pmultiunit	County	US Census American Community Survey 5-Year Data	2018	Percentage (%)
Excluded, non-significant in multivariate model	sv_pnovehicle	County	US Census American Community Survey 5-Year Data	2018	Percentage (%)
Excluded, highly correlated	sv_singleparent	County	US Census American Community Survey 5-Year Data	2018	Percentage (%)
Excluded, non-significant in bivariate model	demo_bridgedrace_p_ american_indians_alaskan	County	CDC, National Center for Health Statistics	2010-2018	Percentage (%)
Excluded, non-significant in bivariate model	demo_bridgedrace_p_ asians_pacific	County	CDC, National Center for Health Statistics	2010-2018	Percentage (%)
Included in final model	demo_bridgedrace_p_ blacks	County	CDC, National Center for Health Statistics	2010-2018	Percentage (%)
Excluded, highly correlated	demo_bridgedrace_p_hisp	County	CDC, National Center for Health Statistics	2010-2018	Percentage (%)

Excluded, highly correlated	demo_bridgedrace_p_whites	County	CDC, National Center for Health Statistics	2010-2018	Percentage (%)
Excluded, highly correlated	demo_bridgedrace_total	County	CDC, National Center for Health Statistics	2010-2018	Percentage (%)
Excluded, highly correlated	days_since_first_case	County	NYT COVID-19 Dataset	Jan 21-Jun 12, 2020	Days

Appendix 4: Descriptive statistics for county variables retained for analysis (median and range for continuous variables)

Variable Code	Training Set (n=1186)		Testing Set (n=593)		Excluded (n=1364)	
como_asthma (%)	9.4	(7.4–12.8)	9.2	(7.4–12.8)	9.2	(7.4–12.8)
como_cancer5yr (cases/5yr)	463.3	(272.1–1135)	457.8	(241–592.1)	451.0	(130.1–677.2)
como_pdiabetes (%)	10.1	(1.5–33.0)	10.0	(1.7–24.6)	9.5	(1.8–32.3)
como_htn_mort (deaths/100,000)	120.2	(20.4–400.6)	120.2	(18.7–442.2)	129.7	(26.5–592.1)
como_medicareheartdizprev (%)	36.0	(22.1–55.2)	35.7	(19.5–49.3)	35.3	(18.0–53.5)
como_smoking (%)	17.3	(9.0–26.8)	17.3	(9.0–26.8)	17.7	(9.0–26.8)
demo_landarea (m^2)	1535.8	(6.5–64008)	1505.1	(38.8–51954)	1743.8	(5.3–377034)
demo_p65more (%)	15.8	(4.2–51.5)	15.6	(6.5–27.6)	19.1	(5.1–36.1)
demo_bridgedrace_p_american_indians_alaskan (%)	0.3	(0.0–73.4)	0.3	(0.1–92.2)	0.5	(0.0–93.9)
demo_bridgedrace_p_asians_pacific (%)	0.9	(0.1–65.5)	1.0	(0.1–30.4)	0.5	(0.0–59.1)
demo_bridgedrace_p_blacks (%)	4.6	(0.1–82.6)	5.5	(0.2–78.5)	1.1	(0.0–85.7)
hc_hospitals (hospitals)	1	(0–32)	1	(0–79)	1	(0–8)
hc_hospitals_per10000 (hospitals/10,000)	0.2	(0.0–3.8)	0.2	(0.0–4.7)	0.4	(0.0–8.5)
hc_icubeds_per60more (beds/>60yr resident)	0.6	(0.0–8.2)	0.7	(0.0–7.0)	0.0	(0.0–101.1)
hc_pnotinsured_acs (%)	9.0	(1.8–39.2)	9.4	(2.0–35.6)	9.3	(1.7–45.6)
hc_primarycare (physicians)	1.9	(0.2–46.6)	1.8	(0.4–17.9)	2.3	(0.2–19.9)
npi_keystone_religious_gatherings_banned (% counties that ban religious gatherings)	52.4		54.8		47.3	
npi_keystone_social_distancing (days since 1st county case)	3	(-46–76)	3	(-30–82)	-8	(-84–46)
ses_punemployed (%)	3.3	(0.5–9.5)	3.3	(0.5–13.6)	2.9	(0.0–16.5)
sv_pcrowding (%)	0.8	(0.0–10.1)	0.9	(0.0–15.4)	0.9	(0.0–35.5)
sv_pmobilehome (%)	9.5	(0.0–54.8)	8.7	(0.0–51.2)	12.2	(0.0–59.3)
sv_pnovehicle (%)	5.8	(1.4–77.0)	5.9	(1.0–32.2)	5.4	(0.0–87.8)

Appendix 5: Regression results

Variable	Exp (Coeff.)	95% CI		Std. Error	Wald	*p*-value	VIF
Intercept	0.0111	(0.0062,	0.0200)	0.2961	-15.2016	<0.0001	
Hospitals per 10,000	0.6773	(0.5549,	0.8248)	0.1013	-3.8485	0.0001	1.0636
Religious Gatherings Ban	0.8752	(0.7894,	0.9702)	0.0526	-2.5315	0.0114	1.0592
Pop. Not Insured (%)	0.9855	(0.9715,	0.9998)	0.0075	-1.9444	0.0519	1.6001
Mobile Home Pop. (%)	0.9921	(0.9854,	0.9989)	0.0035	-2.2539	0.0242	1.7227
Asthma Pop. (%)	1.0951	(1.0400,	1.1533)	0.0264	3.4378	0.0006	1.1489
Pop. >= 65 Yrs. (%)	1.0453	(1.0308,	1.0605)	0.0069	6.4394	<0.0001	1.1405
Total Hospitals in County	1.0316	(1.0100,	1.0522)	0.0099	3.1329	0.0017	1.1663
Black Pop. (%)	1.0097	(1.0063,	1.0133)	0.0018	5.5012	<0.0001	1.2210

County-level predictors of COVID-19 laCFR in the United States, $R^2 = 0.8620$.

Appendix 6: Two variable quantile stratifications

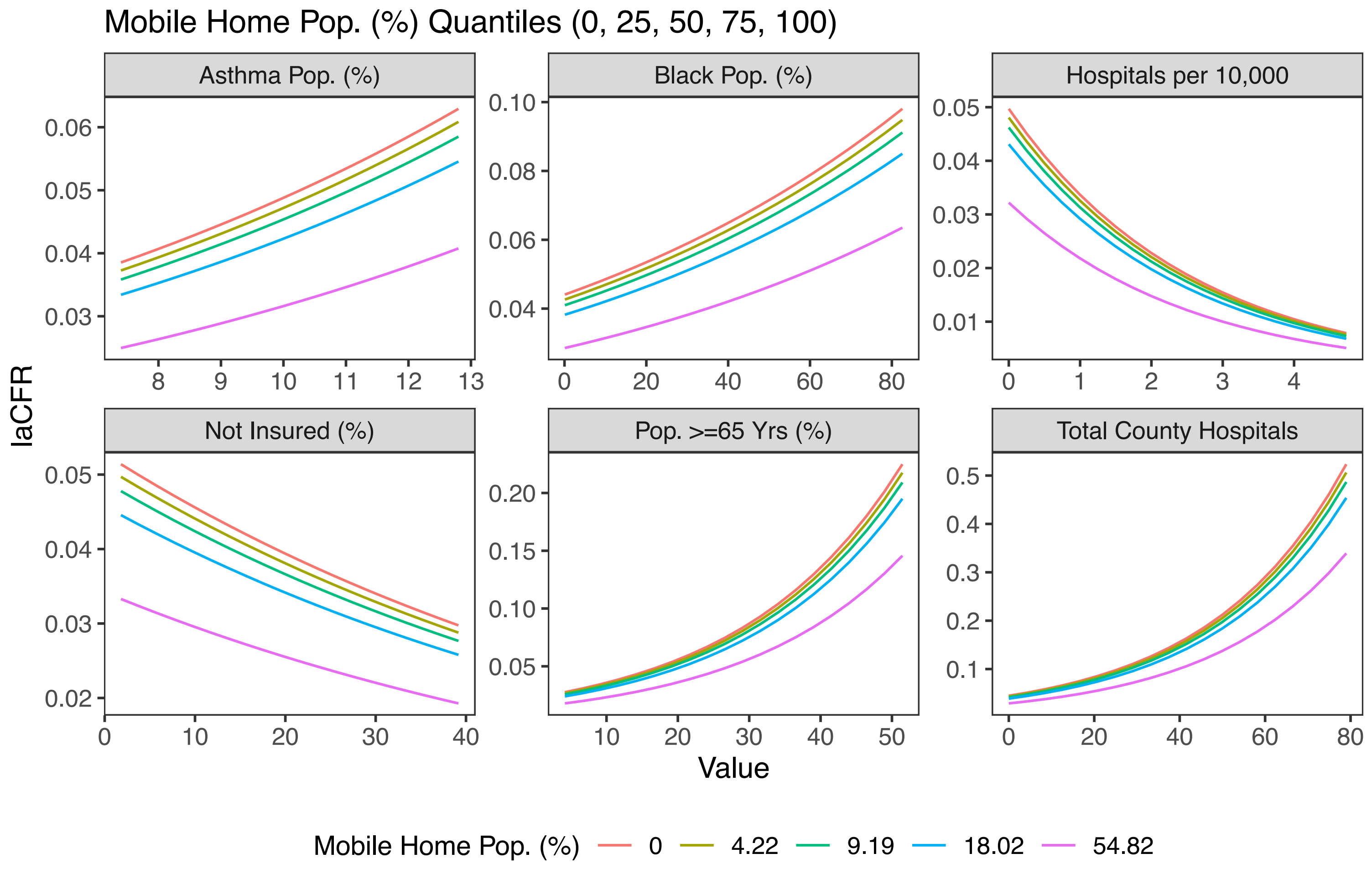

Figure A2. Percentage of county populations living in mobile homes shown as quantiles, over the range of observed values for the second variable. All other variables set at their median (religious gatherings set to 0).

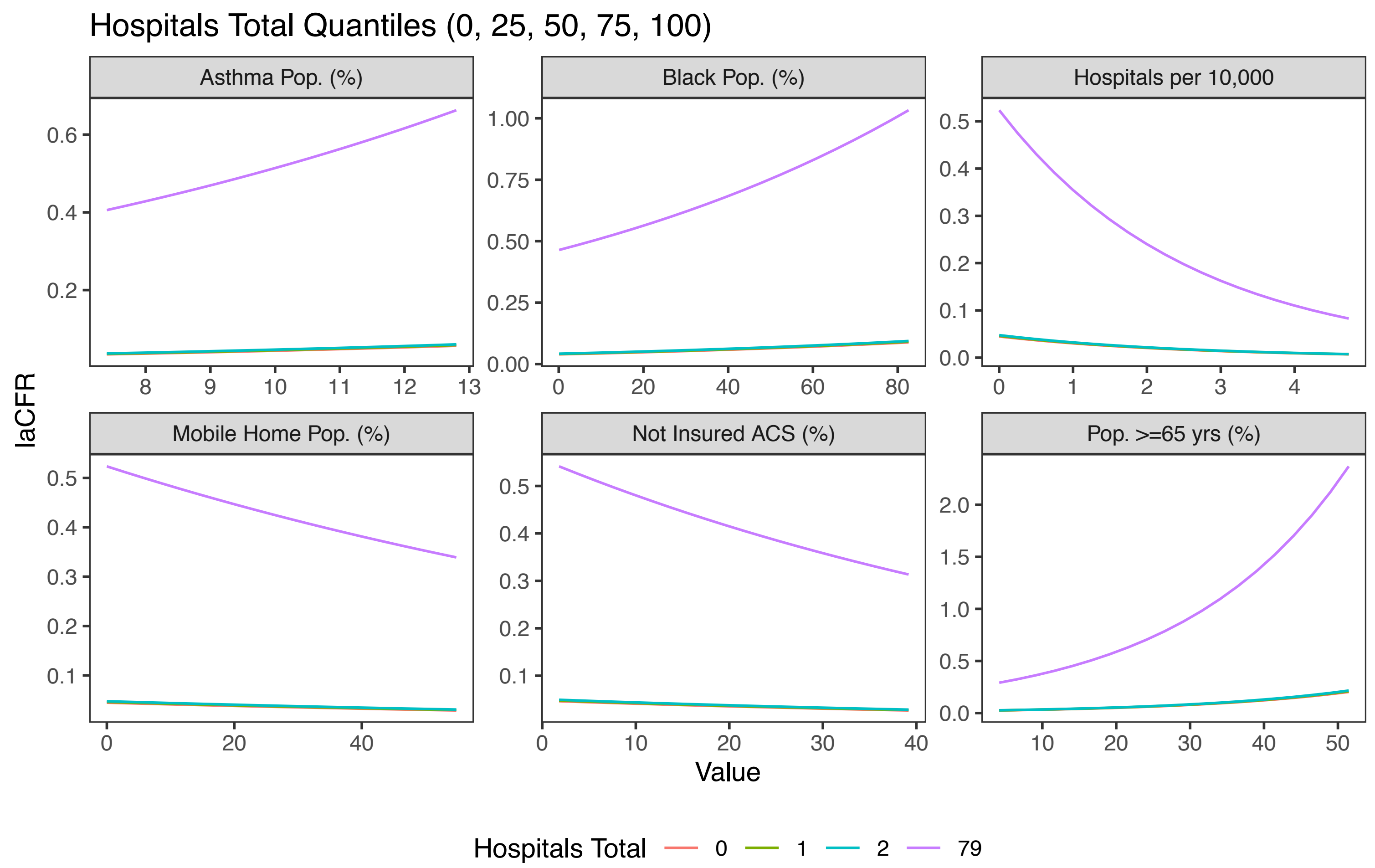

Figure A3. Total number of hospitals within a county shown as quantiles, over the range of observed values for the second variable. All other variables set at their median (religious gatherings set to 0).

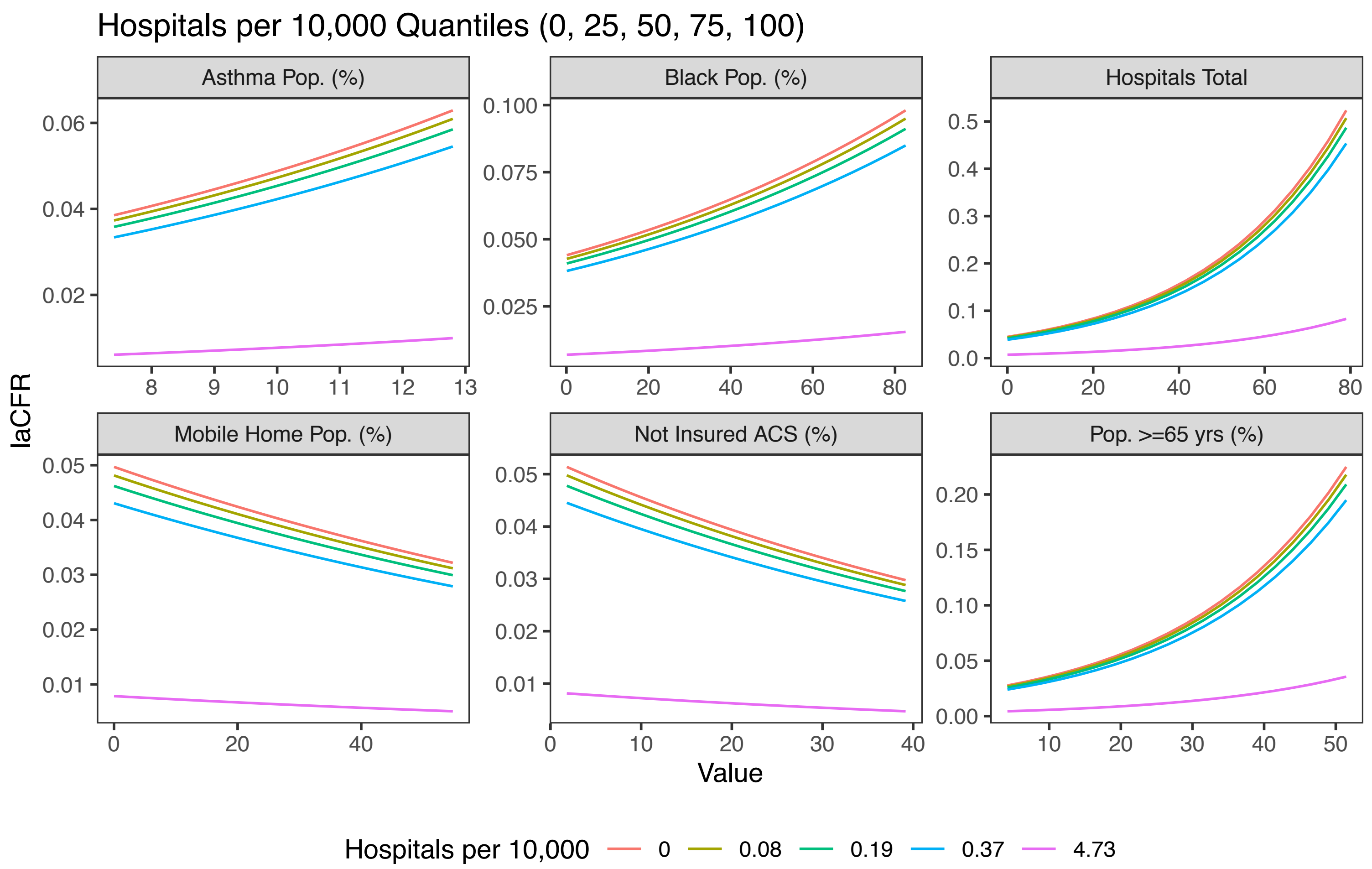

Figure A4. Number of hospitals per 10,000 county residents shown as quantiles, over the range of observed values for the second variable. All other variables set at their median (religious gatherings set to 0).

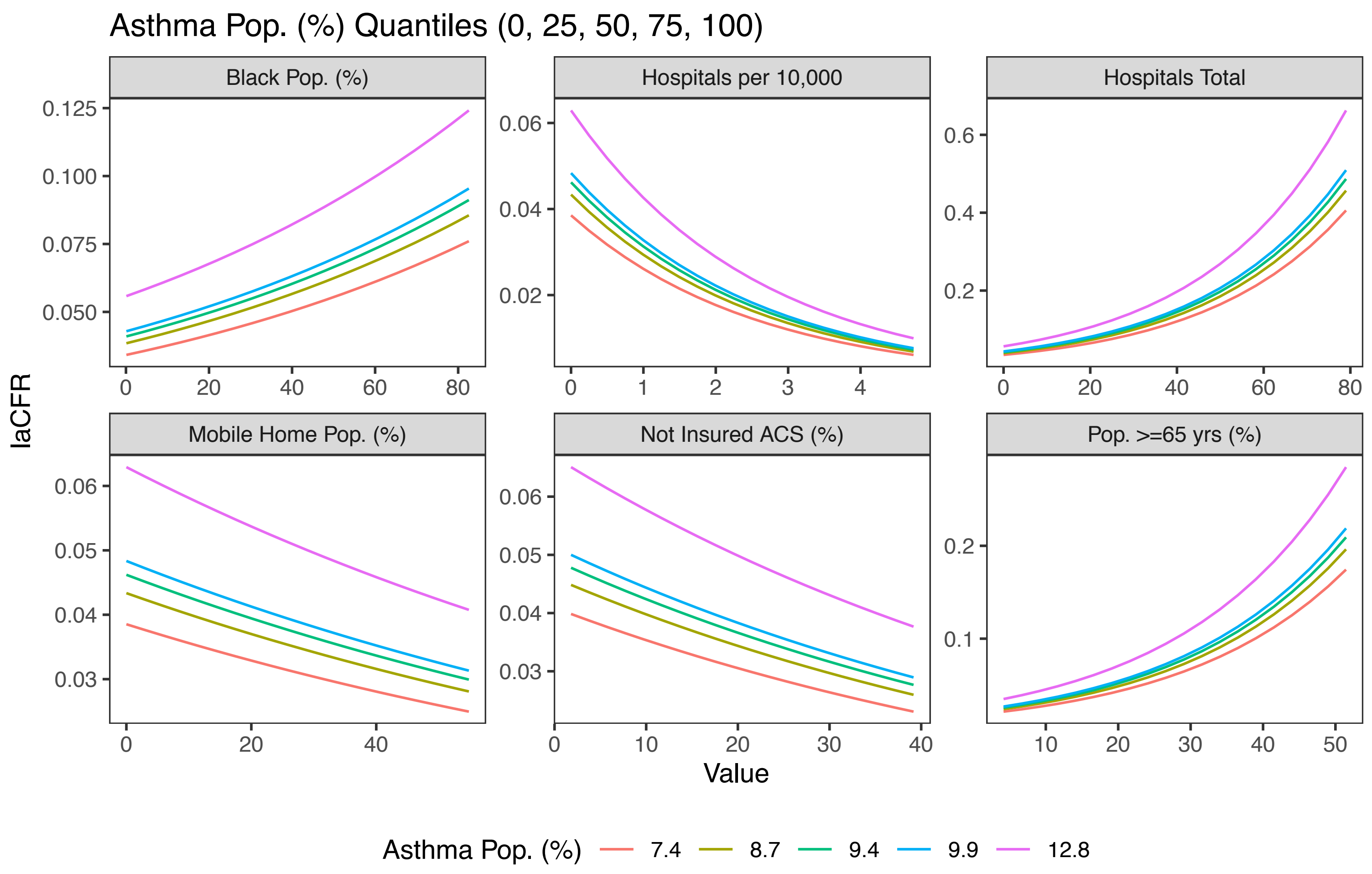

Figure A5. Percentage asthma population within county shown as quantiles, over the range of observed values for the second variable. All other variables set at their median (religious gatherings set to 0).

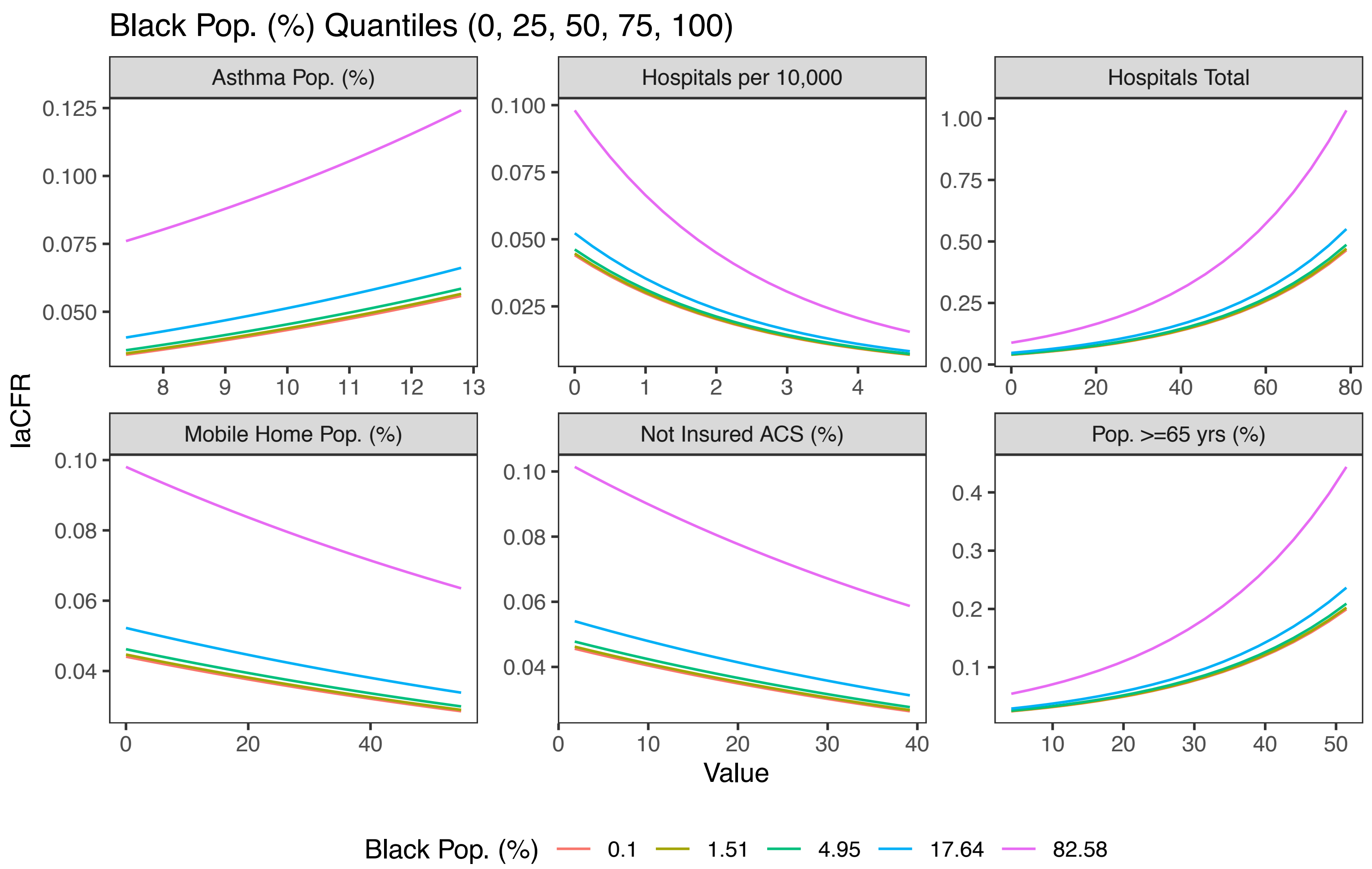

Figure A6. Percentage Black population within county shown as quantiles, over the range of observed values for the second variable. All other variables set at their median (religious gatherings set to 0).

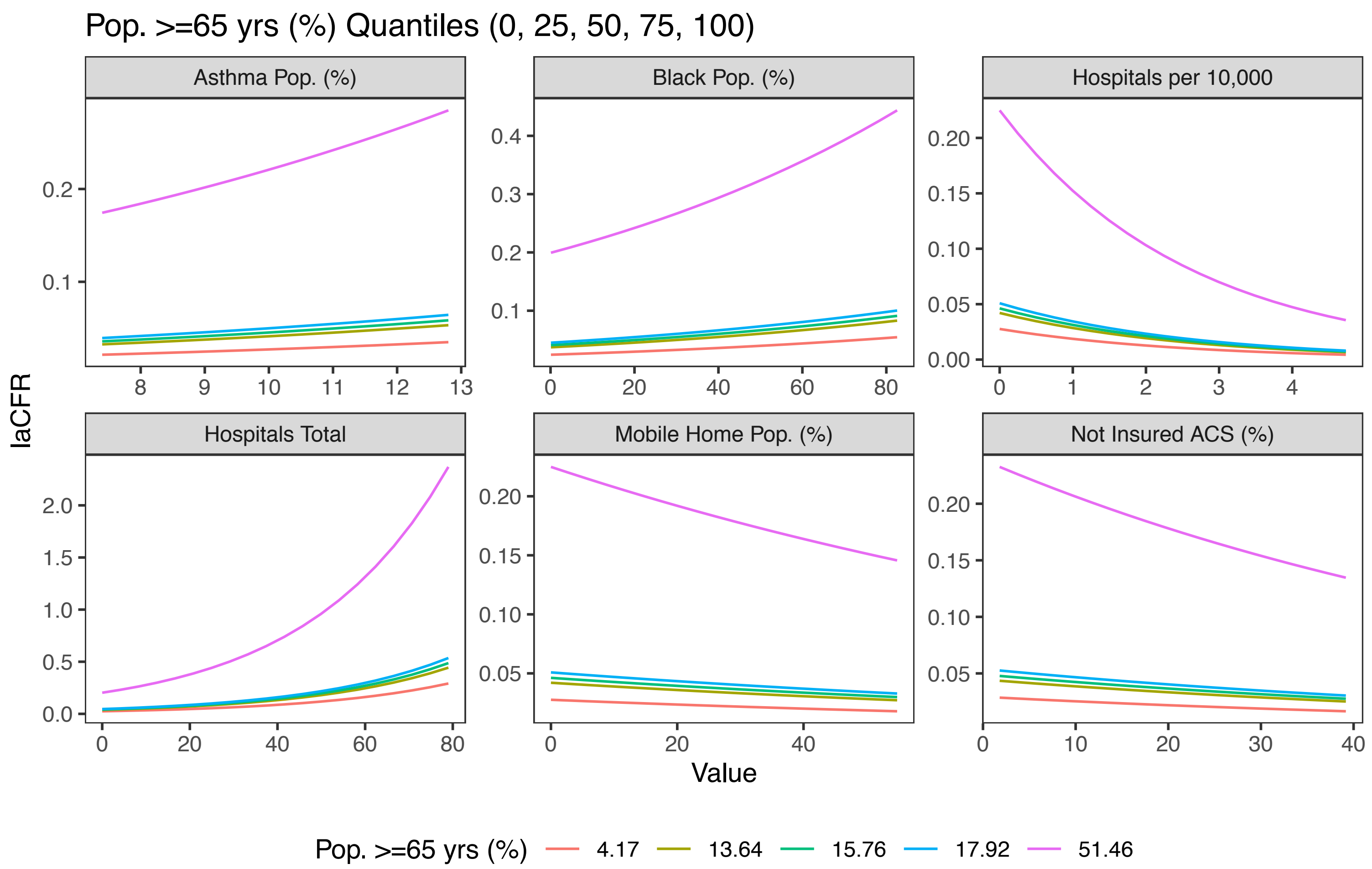

Figure A7. Percentage population 65 years and older within county shown as quantiles, over the range of observed values for the second variable. All other variables set at their median (religious gatherings set to 0).

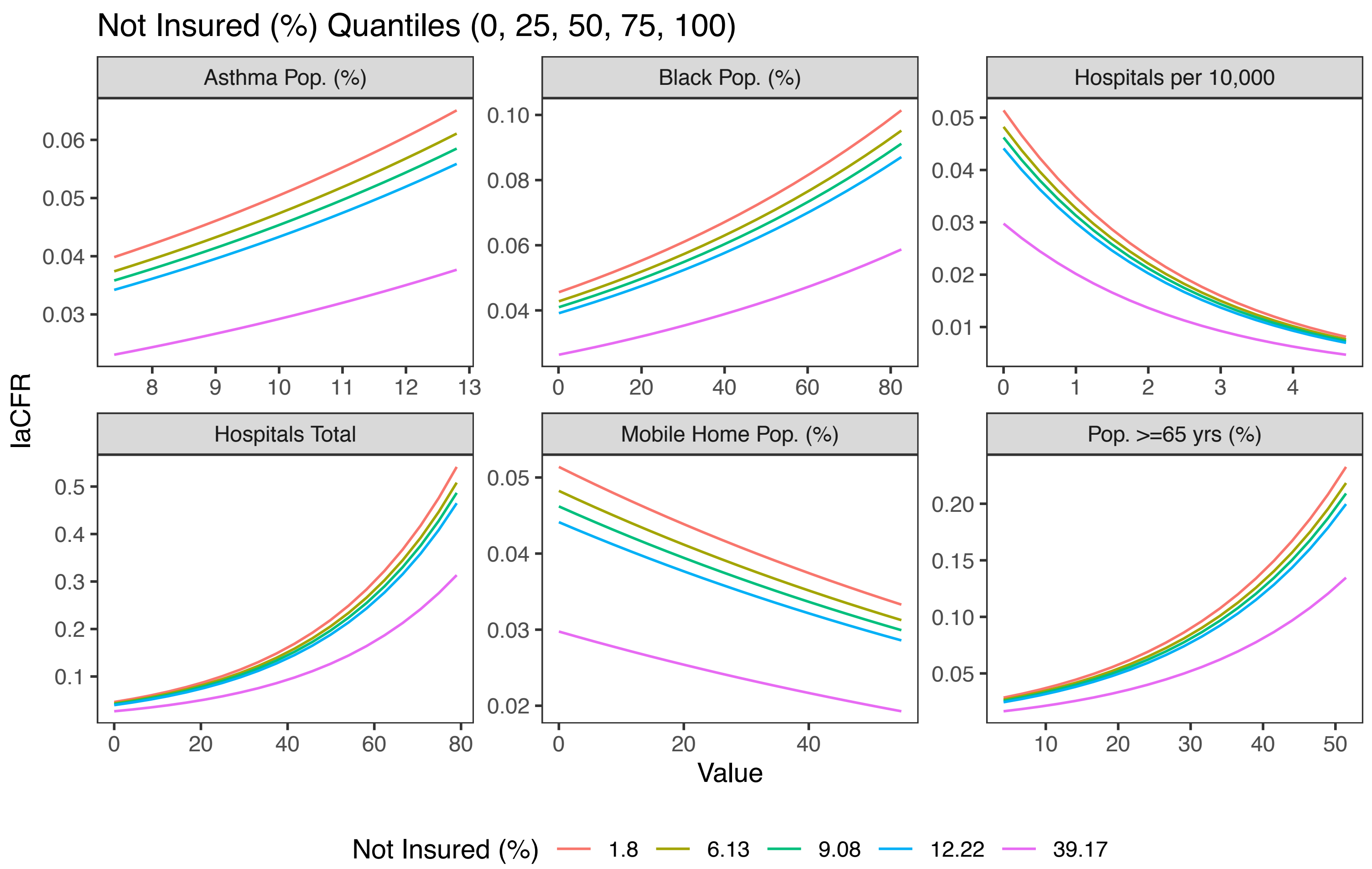

Figure A8. Percentage population 65 uninsured within county shown as quantiles, over the range of observed values for the second variable. All other variables set at their median (religious gatherings set to 0).

Appendix References

1. Bansal M. Cardiovascular disease and COVID-19. Diabetes Metab Syndr. 2020 Mar 25;14(3):247-50.
2. Abrams EM, ’t Jong GW, Yang CL. Asthma and COVID-19. CMAJ. 2020 May 19;192(20):E551.
3. Liang W, Guan W, Chen R, Wang W, Li J, Xu K, et al. Cancer patients in SARS-CoV-2 infection: A nationwide analysis in China. Lancet Oncol. 2020 Feb 14;21(3):335-7.
4. Zhao Q, Meng M, Kumar R, Wu Y, Huang J, Lian N, et al. The impact of COPD and smoking history on the severity of COVID-19: A systemic review and meta-analysis. J Med Virol. 2020 Apr 15;92(10):1915-21.
5. Hussain A, Bhowmik B, do Vale Moreira NC. COVID-19 and diabetes: Knowledge in progress. Diabetes Res Clin Pract. 2020 Apr 9;162:108142.
6. Pranata R, Lim MA, Huang I, Raharjo SB, Lukito AA. Hypertension is associated with increased mortality and severity of disease in COVID-19 pneumonia: A systematic review, meta-analysis and meta-regression. J Renin Angiotensin Aldosterone Syst. 2020 Jun;21(2):1470320320926899.
7. Cai Q, Chen F, Wang T, Luo F, Liu X, Wu Q, et al. Obesity and COVID-19 severity in a designated hospital in Shenzhen, China. Diabetes Care. 2020 May 14;43(7):1392-8.
8. Aggarwal G, Lippi G, Michael Henry B. Cerebrovascular disease is associated with an increased disease severity in patients with Coronavirus Disease 2019 (COVID-19): A pooled analysis of published literature. Int J Stroke. 2020 Apr 20;15(4):385-9.
9. Dobson AP, Carper ER. Infectious diseases and human population history. Bioscience. 1996 Feb; 46(2):115-26.
10. CDC COVID-19 Response Team. Severe Outcomes Among Patients with Coronavirus Disease 2019 (COVID-19) - United States, February 12-March 16, 2020. MMWR Morb Mortal Wkly Rep. 2020 Mar 27;69(12):343-6.
11. Rocklöv J, Sjödin H. High population densities catalyze the spread of COVID-19. J Travel Med. 2020 Mar 29; 27(3):taaa038.
12. Rubinson L, O’Toole T. Critical care during epidemics. Crit Care. 2005 Aug;9(4):311-3.
13. Ayanian JZ, Weissman JS, Schneider EC, Ginsburg JA, Zaslavsky AM. Unmet health needs of uninsured adults in the United States. JAMA. 2000 Oct 25;284(16):2061-9.
14. Wilper AP, Woolhandler S, Lasser KE, McCormick D, Bor DH, Himmelstein DU. Health insurance and mortality in US adults. Am J Public Health. 2009 Dec;99(12):2289-95.
15. Braveman PA, Cubbin C, Egerter S, Williams DR, Pamuk E. Socioeconomic disparities in health in the United States: What the patterns tell us. Am J Public Health. 2010 Apr 1;100(Suppl 1):S186-96.
16. Hahn RA, Truman BI. Education improves public health and promotes health equity. Int J Health Serv. 2015 May 19;45(4):657-78.
17. Hamad R, Elser H, Tran DC, Rehkopf DH, Goodman SN. How and why studies disagree about the effects of education on health: A systematic review and meta-analysis of studies of compulsory schooling laws. Soc Sci Med. 2018 Jul 20;212:168-78.
18. Athar HM, Chang M-H, Hahn RA, Walker E, Yoon P, Centers for Disease Control and Prevention (CDC). Unemployment - United States, 2006 and 2010. MMWR Suppl. 2013 Nov 22;62(3):27-32.

19 Institute of Medicine (US) Committee on Health Care for Homeless People. Dynamics of Homelessness - Homelessness, Health, and Human Needs - NCBI Bookshelf. 1988.
20 Tsai J, Wilson M. COVID-19: A potential public health problem for homeless populations. Lancet Public Health. 2020 Mar 11;5(4):e186-7.
21 Sullivan WF. Briefing Note: Primary Care Guidance Regarding COVID-19 for Those Who Care for Adults with Intellectual and Developmental Disabilities [Internet]. Toronto, ON, Canada: Surrey Place; 2020 [cited 2020 Aug 30]. Available from: https://www.surreyplace.ca/wp-content/uploads/2020/04/COVID-19-Guidance-for-Prevention-and-Care.pdf.
22 Baker MG, McDonald A, Zhang J, Howden-Chapman P. Infectious diseases attributable to household crowding in New Zealand: A systematic review and burden of disease estimate. Wellington: He Kainga Oranga/Housing and Health Research; 2013.
23 Bonini A, Mukherjee S, Roig M. Responses to the COVID-19 catastrophe could turn the tide on inequality [Internet]. United Nations Department of Economic and Social Affairs; 2020 May [cited 2020 May 18]. Available from: https://www.developmentaid.org/api/frontend/cms/uploadedImages/2020/05/PB-65.pdf.
24 Armitage R, Nellums LB. The COVID-19 response must be disability inclusive. Lancet Public Health. 2020 Mar 27;5(5):e257.
25 Shi L, Lebrun LA, Tsai J. The influence of English proficiency on access to care. Ethn Health. 2009 Dec;14(6):625-42.
26 Wimalawansa SJ. Global Epidemic Of Coronavirus—Covid-19: What Can We Do To Minimize Risks. European Journal of Biomedical. 2020;7(3):432-8.
27 Bayham J, Fenichel EP. Impact of school closures for COVID-19 on the US health-care workforce and net mortality: A modelling study. Lancet Public Health. 2020 Apr 3;5(5):e271-8.
28 Feagin J, Bennefield Z. Systemic racism and U.S. health care. Soc Sci Med. 2014 Feb;103:7-14.
