# Supplementary material for: Risk factors for increased COVID-19 case-fatality in the United States: A county-level analysis during the first wave
Source: PLoS One. 2021 Oct 14;16(10):e0258308. doi: 10.1371/journal.pone.0258308 (PMC8516194; doi:10.1371/journal.pone.0258308)
Supplement: Attachment — Submitted filename: PLoS-reviewer-comments.docx [file pone.0258308.s002.docx]

Dear Editor,

Thank you for the opportunity to revise and resubmit our manuscript. Below is a point-by-point response to the reviewer comments. Addressing these comments has significantly strengthened our manuscript, and we look forward to next steps in the review process.

**Reviewer #1, comment 1**: “By June 12th, 2020, SARS-CoV-2 had caused over 2 million Coronavirus Disease 2019 (COVID-19) cases and 114,753 deaths in the United States (US) (2,3)” please update this data

**Response**: Thank you for this comment. We opted to use this date as our work only considered cases during the first wave of the pandemic in the U.S. (i.e., through June 12, 2020). We have clarified this point and added additional information on the burden of COVID-19 in lines 49–50.

**Reviewer #1, comment 2**: The Research hypotheses and objective need to be clear in the introduction section

**Response**: Thank you for letting us know that this was unclear. We have rephrased the last paragraph of the introduction (lines 74, 76–78) to highlight that our study objective was to use a lag-adjusted case fatality rate (laCFR) to determine which population-level variables were most associated with differences in laCFR across U.S. counties.

**Reviewer #1, comment 3**: Study Population, Research flow chart needs to be provided. How many patients included and excluded, why?

**Response**: Thank you for this helpful suggestion. As this study was ecological in design, we did not enroll individual patients. Instead, our unit of analysis was U.S. counties. We have therefore added a flowchart in our supplemental materials indicating our county inclusion process (Appendix 1). Of 3,413 total U.S. counties, 3,004 counties had experienced at least one COVID-19 case by the end of our study window, June, 12, 2020. Only 1,779 counties reached a stable laCFR by the end of our study window. These counties were split into a training set (1,186 counties) and a testing set (593 counties).

**Reviewer #1, comment 4**: How potential risk factors were identified? Why those factors were included.

**Response**: Thank you for raising this need for clarification. Our process of variable inclusion had 2 steps: (1) an identification of the initial cohort of variables and (2) the variable selection for the regression model. For the first step, we identified potential county-level risk factors through a comprehensive literature review, which we further supplemented with variables relevant to other respiratory epidemics. In our original submission, we provided a detailed description and justification for the inclusion of the initial cohort of variables in Appendices 2–4. For the second step, as described in the main text of our original submission, we removed highly correlated variables to reduce multicollinearity and used purposeful selection to identify variables to be kept in the regression model. Since the first step was not clearly described in the main text, we have edited and added more details on the inclusion of these variables in the Methods section (lines 101–106).

**Reviewer #1, comment 5**: Results, “retained variables, neither of which appeared in the final model (21)” reference should not be included in the results section.

**Response**: Thank you for catching this oversight. We have moved the citation to the Methods section (line 127).

**Reviewer #1, comment 6**: a county-level mortality could be compared between March 28, 2020 to June 12, 2020 vs. March 28, 2019 to June 12, 2019?

**Response**: Thank you to the reviewer for this interesting suggestion on examining excess mortality at the county level. Unfortunately, our work relies on publicly available data sources and complete data on all-cause mortality for 2020 is not yet available from the National Vital Statistics System (i.e., the gold standard source for mortality data in the U.S.) at time of writing this response letter. As such, an excess mortality analysis comparing 2019 and 2020 is not possible in the present work. We will keep this suggestion in mind for future work, as sufficiently complete mortality data become publicly available.

**Reviewer #1, comment 7**: What is the clinical utility of the authors findings? The clinical perspective should be confirmed. The findings could be used to reduce the COVID-19 case-fatality in the United States?

**Response**: We would like to thank the reviewer for reminding us of the importance of a clinical perspective during the ongoing pandemic. Notably, this work is not designed to provide insight to clinicians seeking to care for individual patients; instead, this work uses an ecological study design and therefore considers the laCFR in a county as whole, without linking specific cases and deaths to specific individuals. Such a design is an excellent approach for public (i.e., population-level) health problems, however––and as a result, our work aims to provide decision-making support for public health officials who seek to understand which counties are most at risk of high rates of COVID-19 deaths. Investing in public health in such a way helps individual patients by informing targeted resource deployment such as vaccines, non-pharmaceutical interventions, and extra healthcare workers and supplies to the counties that need them most.

**Reviewer #1, comment 8**: In Discussion section, the authors mainly summarized again their observations and how they were similar or different from prior studies. This reviewer would expect to see some points regarding how to translate these observations to help address this public health concern.

**Response**: Thank you for this suggestion. As this paper is not focused on the effectiveness of specific interventions, we sought to explain reasons why we observed the associations we did without going beyond the bounds of what our work reasonably allows us to infer. In the Discussion section, we sought to contextualize our results but also felt it was important to avoid making specific policy recommendations that could not be supported directly from the present work. Therefore, to contextualize our results, we presented possible reasons for each of the observed relationships, supported by additional literature, especially when our results differed from the results seen in individual-level studies. Our finding that more hospitals per 10,000 persons were associated with lower laCFR is one example of such a result. In the Discussion section, we connect this finding to several possible reasons why this might be so: increased availability of hospital beds, increased ability to expand capacity rapidly, increased community wealth, and increased competition between hospitals leading to better outcomes for patients. Because our study was not designed to test which of these possible reasons caused the observed association, it would be an overstatement to make policy recommendations based on this observation. We faced a similar dilemma for each of the variables included in our model, so we opted to present well-researched potential reasons for their association with laCFR without providing specific recommendations. Instead, we suggest that these are factors worth considering but leave policy decisions to stakeholders to avoid overstating our findings.

**Reviewer #1, minor comment**: Added the following references in the revision text “Tu, WJ., Cao, J., Yu, L. et al. Clinicolaboratory study of 25 fatal cases of COVID-19 in Wuhan. Intensive Care Med 46, 1117–1120 (2020). https://doi.org/10.1007/s00134-020-06023-4; Cao, J., Tu, W. J., Cheng, W., Yu, L., Liu, Y. K., Hu, X., & Liu, Q. (2020). Clinical features and short-term outcomes of 102 patients with coronavirus disease 2019 in Wuhan, China. Clinical Infectious Diseases, 71(15), 748-755.”

**Response**: Thank you for this suggestion. We have added these citations to the introduction (lines 54–57).

**Reviewer #2, only comment**: This manuscript does not bring new useful information to readers, so it may not get more attention from readers. The manuscript is less innovative. It is recommended to add relevant analysis indicators and data to improve the quality.

**Response**: Thank you to the reviewer for taking the time to read our work. We respectfully disagree with this assessment, and further feel that our work is scientifically sound and therefore eligible for publication in PLoS One. We believe that the careful consideration of factors associated with COVID-19 laCFR at the population level is innovative and of interest to public health officials, particularly because the factors previously associated with risk of COVID-19 at the individual level did not always match the factors identified in our model at the population level. Given that both clinical (i.e., individual-level) and public health (i.e., population-level) decision-making is essential to mitigating a pandemic, thus necessitating both types of analyses. We would gladly incorporate additional information to strengthen our paper, as we have sought to do for the other reviewer comments, but we were unable to determine what additional indicators and data were viewed as necessary to improve this work from the feedback provided here.

**Reviewer #3, comment 1**: The first wave of the COVID-19 pandemic defined from March 28, 2020 to June 12, 2020.But how is the first wave of a pandemic defined. The reasons given in the article do not seem particularly convincing.

**Response**: Thank you for letting us know that we did not provide adequate support for our selection of dates for the first wave. To address this concern, we have added a citation (lines 358–360) to a paper that discusses the three waves of the COVID-19 pandemic, a spring 2020 wave, a summer 2020 wave, and a fall/winter 2020/2021 wave. The paper does not give exact start and end dates for the waves, but Figure 1a plots cases over time and it appears that the first “spring” wave begins towards the end of March 2020 and ends at some point in mid-June 2020. We hope that this additional citation, combined with the other reasons previously presented in our manuscript, adequately defends our choice of dates.

**Reviewer #3, comment 2**: Some variables can be further stratified for comparison. The more detailed analysis of these variables might lead to more precise conclusions.

**Response**: Thank you for this suggestion for stratification. We have added the stratification results in the Appendix A9 and have also mentioned them in the Results section (lines 172–173).

**Reviewer #3, comment 3**: Some of the images should be further simplified and beautified. It can better improve the readability of the paper.

**Response**: Thank you for pointing this out. After careful consideration of our figures, we determined that Figure 1 was the figure that needed simplification and beautification. We have therefore split what was Figure 1 into two separate figures, to allow space for clearer legends. We are happy to make additional changes but felt that the remaining figures were already sufficiently simple and clear.

**Reviewer's Responses to Question 3**: Have the authors made all data underlying the findings in their manuscript fully available?

Reviewer #1: Yes

Reviewer #2: No

Reviewer #3: Yes

**Response**: For the purposes of reproducibility, all data, code, and model calibration specifications (i.e., which seeds were used, etc.) for our paper were deposited in our GitHub repo (https://github.com/jmillar201/COVID19_CFR) at the time of our original submission.

Thank you for your time and consideration,

The Authors
