# Supplementary material for: Risk factors for increased COVID-19 case-fatality in the United States: A county-level analysis during the first wave
Source: PLoS One. 2021 Oct 14;16(10):e0258308. doi: 10.1371/journal.pone.0258308 (PMC8516194; doi:10.1371/journal.pone.0258308)
Supplement: Attachment — Submitted filename: PLoS-reviewer-comments2.docx [file pone.0258308.s003.docx]

Dear Editor,

Thank you for the opportunity to revise and resubmit our manuscript. Below is a point-by-point response to the reviewer comments. Addressing these comments has significantly strengthened our manuscript, and we look forward to next steps in the review process.

**Editor, comment 1**: Please review your reference list to ensure that it is complete and correct.

**Response**: We have checked all of the citations and replaced two preprints (details below). We have also provided archived links for several non-journal citations (such as research from the CDC) to make sure these links are stable over time. Here is the reasoning for citations that are not journal articles or book chapters:

Citation 2:

Citing where COVID19 cases and death data came from

Citation 4:

Citing where estimation of total mortality due to COVID-19 is located

Citation 13:

Citing our code that we used for this project on GitHub

Citation 17:

Citing where county level race data was acquired

Citation 18:

Citing where county level ICU beds and percent insured data was acquired

Citation 21-23:

Citation of work being done with the CDC on social vulnerability

Citation 25:

Citing where county level ICU bed data was acquired

Citation 26:

Citing where county level heart disease data was acquired

Citation 27:

Citing where county level diabetes data was acquired

Citation 28:

Citing where county level smoking and asthma data was acquired

Citation 29:

Citing where county level cancer data was acquired

Citation 30:

Citing where county level COVID19 non pharmaceutical intervention data was acquired

Citation 31:

Citing CDC reopening guidelines

Citation 32:

Citing where New York City county level COVID19 cases and death data came from

Citation 41:

This was a preprint citation that covered gain curve plots and their uses pretty well. Since this is a preprint, we went ahead and replaced this citation with a book chapter instead, covering the same topic

Citation 48:

Citing a policy brief released by the Community and Labor Center on the effect of COVID-19 on workers

Citation 54:

Citing National Center of Health Statistics on risk for age related mortality due to COVID-19

Citation 56:

Citing CDC on risk for asthma related mortality due to COVID-19

Citation 60:

Citing National Association of Counties (organization that represents county governments) on effects of structural racism in the US

Citation 68:

Citing news article on how Florida was found to report fewer COVID-19 deaths in the official tally than the Medical Examiners Commission

Citation 71:

This was a preprint citation that covered the different waves of the pandemic. Since this is a preprint, we have replaced this with a different article that is published

Citation 72:

Citation covering the shift in reporting to the HHS Protect system, as of June 12th

**Editor, comment 2**: Please be clear about the innovative nature of this research.

**Response**: Thank you for letting us know that this was unclear. We have expanded upon the innovative nature of the paper in the introduction. This work expands upon prior work by considering possible risk factors of increased mortality from multiple categories at once (e.g., non-pharmaceutical interventions such as shelter-in-place orders, prevalence of pre-existing conditions such as cardiovascular disease, and socio-economic circumstances such as hospital accessibility) in a single model. This is also the first paper to focus on this range of risk factors during the first wave of the pandemic, so that results from this can be used for targeted intervention at the county level at the beginning of a pandemic.

**Reviewer #2, comment 1**: Is the manuscript technically sound, and do the data support the conclusions?: Partly

**Response**: Reviewer 2 felt that either our manuscript was not technically sound or that the data do not support the conclusions we drew. However, reviewer 2 provided no comment on what the remaining issues were. We do feel our work is sound and our conclusions were appropriate, so are uncertain what to do to resolve this issue.

Thank you for your time and consideration,

The Authors
